# Negative Emotion Differentiation Attenuates the Within-Person Indirect Effect of Daily Stress on Nightly Sleep Quality Through Calmness

**DOI:** 10.3389/fpsyg.2021.684117

**Published:** 2021-08-11

**Authors:** Tanja Lischetzke, Lea Schemer, Julia A. Glombiewski, Tina In-Albon, Julia Karbach, Tanja Könen

**Affiliations:** Department of Psychology, University of Koblenz-Landau, Landau, Germany

**Keywords:** negative emotion differentiation, negative emotional granularity, daily stress, stress reactivity, calmness, sleep quality, COVID-19, multilevel mediation analysis

## Abstract

The ability to differentiate between negative emotional states [negative emotion differentiation (NED)] has been conceptualized as a trait that facilitates effective emotion regulation and buffers stress reactivity. In the present research, we investigated the role of NED in within-person processes of daily affect regulation and coping during times of stress (the first COVID-19-related pandemic lockdown in April 2020). Using intensive longitudinal data, we analyzed whether daily stress had an indirect effect on sleep quality through calmness in the evening, and we tested whether NED moderated this within-person indirect effect by buffering the link between daily stress and calmness in the evening. A non-representative community sample (*n* = 313, 15–82 years old) participated in a 21-day ambulatory assessment with twice-daily surveys. The results of multilevel mediation models showed that higher daily stress was related to within-day change in calmness from morning to evening, resulting in less calmness in the evening within persons. Less calmness in the evening, in turn, was related to poorer nightly sleep quality within persons. As expected, higher NED predicted a less negative within-person link between daily stress and calmness in the evening, thereby attenuating the indirect effect of daily stress on nightly sleep quality through calmness. This effect held when we controlled for mean negative emotions and depression. The results provide support for a diathesis-stress model of NED, and hence, for NED as a protective factor that helps to explain why some individuals remain more resilient during times of stress than others.

## Introduction

Individual differences in emotion differentiation (also called emotional granularity) refer to the degree to which individuals make fine-grained distinctions between similarly valenced emotional states (Barrett et al., 2001; Tugade et al., 2004). Individuals high in emotion differentiation tend to use discrete emotion words (e.g., angry, disappointed, and lonely) in a context-specific way, whereas individuals low in emotion differentiation tend to use same-valenced emotion words interchangeably across different situational contexts. In particular, the ability to differentiate between negative emotional states [negative emotion differentiation (NED)] has been conceptualized as a trait that facilitates effective emotion regulation and thereby promotes well-being (e.g., Kashdan et al., 2015). Two recent meta-analyses demonstrated a significant but small association between NED and psychosocial functioning: The results by O’Toole et al. (2020) indicated a small positive relation between NED and behavioral adaptation in non-clinical populations, and the results by Seah and Coifman (2021) indicated a small negative association between NED and the enactment of maladaptive behaviors, such as aggression or avoidance. The fact that the meta-analytic effect sizes were rather small may call into question the importance of NED as an adaptive skill. However, as O’Toole et al. (2020) and others (e.g., Barrett et al., 2001; Kashdan et al., 2015; Ottenstein and Lischetzke, 2020) have argued, high NED can be assumed to be most helpful under circumstances that evoke intense negative emotions (e.g., stressful events). In the present study, we aimed to shed more light on the assumed adaptive value of NED during times of stress.

Negative emotion differentiation is typically measured indirectly in daily life and operationalized as the degree of covariation between negative emotions over time (Erbas et al., 2014). That is, individuals are requested to repeatedly rate their momentary emotional experience using ambulatory assessment (AA) methodology (also termed experience sampling or ecological momentary assessment; Trull and Ebner-Priemer, 2014). For each individual, the degree of covariation between negative emotions over time is quantified by the intraclass correlation coefficient (ICC) measuring average consistency. A high ICC reflects that individuals frequently report feeling different discrete negative emotions (such as anger, sadness, or fear) at the same time (i.e., low NED). A low ICC reflects that individuals report more divergent patterns of negative emotional experience depending on the circumstances (i.e., high NED).

A large portion of previous research on NED can be classified into two major types of studies: The first major type of study compared NED in healthy controls and clinical populations, including individuals diagnosed with major depressive disorder (Demiralp et al., 2012), social anxiety disorder (Kashdan and Farmer, 2014), borderline personality disorder (Suvak et al., 2011), schizophrenia (Kimhy et al., 2014), and autism spectrum disorder (Erbas et al., 2013). Taken together, the findings from these studies indicated that low NED might represent a transdiagnostic factor that contributes to the development and maintenance of various mental disorders.

In the second type of study, concurrent or predictive associations of NED with other individual difference constructs (e.g., measured via global self-report or estimated via aggregated/mean repeated states) were analyzed. Among the individual differences that have been studied were emotional clarity (Boden et al., 2013), verbal ability (Ottenstein and Lischetzke, 2020), emotion regulation (Barrett et al., 2001; Ottenstein, 2020), emotional intelligence (MacCann et al., 2020), mindfulness (Tong and Keng, 2017), physical health (Oh and Tong, 2020), psychopathological symptoms (Liu et al., 2020; Schreuder et al., 2020), and well-being (Lennarz et al., 2018; Dejonckheere et al., 2019; Ottenstein, 2020). This type of research has helped to map out the nomological net that reflects the potential antecedents and consequences of NED. However, the added value of NED (and other emotional complexity measures) in predicting overall levels of well-being and psychopathology has recently been called into question because the predictive utility of NED disappeared when mean affect was controlled for (Dejonckheere et al., 2019, Schreuder et al., 2020). This allows two conclusions: First, it is important to test whether the predictive utility of NED remains significant after accounting for the mean levels of negative emotions. Second, more research is needed on the role that NED plays in predicting *individual differences in within-person processes* of momentary affect regulation and daily coping with stress. Thereby, process-oriented studies might shed more light on why low NED is related to higher overall levels of negative emotionality and a higher risk of developing psychopathological symptoms.

To date, only relatively few studies have investigated NED as a predictor of individual differences in within-person affect-related processes. In one AA study (Kashdan et al., 2010), NED moderated (buffered) the within-person link between momentary negative affect and alcohol consumption, and in another AA study (Pond et al., 2012), NED buffered the within-person link between momentary anger and aggressive behavior. Recently, Starr et al. (2020) proposed a diathesis-stress model of NED. They hypothesized that individuals with high (vs. low) NED may be “better prepared to manage the emotional and behavioral aftermath of stress exposure” (p. 2), decreasing the likelihood that stressful experiences result in depressive symptoms. In a similar vein, Kashdan et al. (2015) argued that high differentiators may be less likely to be overwhelmed in stressful situations. Consistent with Starr et al.’s diathesis-stress model, NED moderated the within-person relation between daily hassles and daily depressed mood in a community sample of adolescents: For low differentiators, daily hassles were more strongly associated with higher daily depressed mood than for high differentiators (Starr et al., 2020). Starr et al. (2017) also found that NED moderated the within-person relation between daily hassles and daily depressed mood in a sample of help-seeking veterans. However, this moderator effect did not generalize to a sample of college students—which suggests that further replication of the proposed stress-buffering effect of NED is warranted. In the present research, we sought to conceptually replicate Starr et al. (2017, 2020) findings in the context of coping with stressors during times of crisis by testing whether NED would be found to buffer the link between daily stress and calm-tense mood in the evening.

**FIGURE 1:**
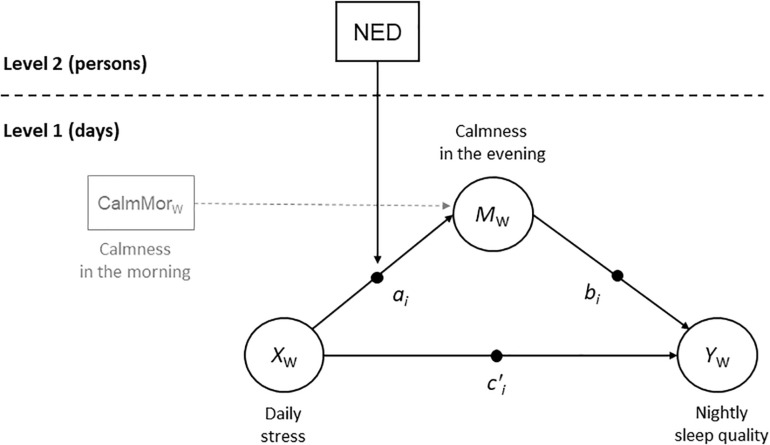
Moderated multilevel (1-1-1) mediation model (First-Stage Multilevel Conditional Process Model). Index W indicates the within-person part of the time-varying variables. Time was included as a control variable at Level 1 (predicting M_W_ and Y_W_), but for simplicity, time is not depicted. NED, negative emotion differentiation.

Moreover, we aimed to extend the within-person process under scrutiny by additionally analyzing the potential detrimental consequences of tense mood on sleep quality. We expected an indirect within-person effect of perceived daily stress on subjective sleep quality through calmness in the evening (see the Level 1 part of Figure 1). Within-person fluctuations in daily stress have been shown to be associated with fluctuations in nightly sleep quality: In healthy adults (Morin et al., 2003; Garde et al., 2011; Åkerstedt et al., 2012; Tousignant et al., 2019) and in individuals with insomnia (Morin et al., 2003), subjective sleep quality was lower on the days on which individuals experienced more stress than usual. Heightened cognitive and somatic arousal before bedtime have been proposed as mediators of the link between daily stress and sleep quality (Morin et al., 2003; Winzeler et al., 2014; Tousignant et al., 2019). Consistent with this view, a recent review that summarized findings of AA studies on the within-person link between day-to-day fluctuations in sleep and mood (Konjarski et al., 2018) suggested that feelings of serenity and calmness (i.e., low tense arousal) are the most beneficial feelings for a good night’s sleep. In the present research, we aimed to test whether daily stress has an indirect effect on sleep quality through calmness in the evening. On the basis of the diathesis-stress model of NED by Starr et al. (2017, 2020), we expected that NED would moderate this within-person indirect effect by buffering the link between daily stress and calmness in the evening (see Figure 1).

What might be the mechanisms by which higher NED ameliorates the adverse impact of daily stress on mood? Drawing on theoretical accounts of NED and stress management, Starr et al. (2020) proposed that high (vs. low) differentiators should be better able to identify the cause of their experienced emotions in response to stressors, and hence, to generate an adaptive response. Similarly, Kashdan et al. (2015) speculated that high NED should make it easier for individuals to shift their attentional focus and adopt a more self-distanced perspective on their feelings, thereby enhancing the opportunity for goal-directed regulatory behavior. On the basis of these (yet untested) ideas, we additionally aimed to explore whether daily rumination about emotions would increase daily stress reactivity and whether low NED would predict more daily rumination. In one of two studies, Kalokerinos et al. (2019) found empirical support that lower NED predicted more rumination in daily life. However, given that rumination was operationalized as referring to a single specific event in this study (first-year students receiving grades) and that the evidence was inconsistent, more research is warranted.

## The Present Research

With the present research, we aimed to conceptually replicate and extend Starr et al.’s (2017, 2020) findings on the stress-buffering effect of NED. We conducted a 3-week AA study during times of stress (the first pandemic lockdown in 2020) to investigate the indirect within-person effect of perceived daily stress on nightly subjective sleep quality through calmness in the evening. We selected tense vs. calm mood as mood dimension of interest because it has been conceptualized as an indicator of psychological stress reactivity (e.g., Klaperski et al., 2013) and was positively related to nightly sleep quality (Konjarski et al., 2018) and negatively related to depressive symptoms (e.g., Huffziger et al., 2013; Timm et al., 2017). We tested the following hypotheses:

*Hypothesis 1:* Daily stress will have an indirect effect on poorer subjective sleep quality through calmness in the evening.

*Hypothesis 2:* The within-person indirect effect of daily stress on nightly sleep quality through calmness in the evening will vary on the basis of NED. Specifically, NED will moderate the within-person relation between daily stress and calmness in the evening such that at higher (vs. lower) levels of NED, higher daily stress will be less strongly associated with a more tense mood in the evening.

In statistical terms, these hypotheses translate into a first-stage multilevel conditional process model (Hayes and Rockwood, 2020)—that is, a moderated multilevel (1-1-1) mediation model in which the predictor *X* (daily stress), the mediator *M* (calmness in the evening), and the dependent variable *Y* (that night’s sleep quality) are measured at Level 1 (i.e., the day level), and NED is included as a Level 2 (i.e., person-level) moderator of the Level 1 association between *X* and *M* (cross-level interaction).

To control for potential effects of day-to-day fluctuations in mood on the perception of daily stress, we assessed calmness in the morning and included it as an additional Level 1 predictor of calmness in the evening in the multilevel mediation model. This allowed us to model within-day change in calmness from morning to evening, and hence, path *a*_*i*_ in the multilevel (1-1-1) mediation model (see Figure 1) represented the within-person relation between daily stress and within-day change in calmness (for a similar strategy, in which lagged affect is included as a Level 1 predictor, see, e.g., Kalokerinos et al., 2019). To test whether the hypothesized moderating effect of NED would hold when controlling for mean negative affect (Dejonckheere et al., 2019; Schreuder et al., 2020), we added individuals’ mean level of negative emotions across the AA study phase and their level of depressive symptoms (assessed shortly before the start of the AA study phase) as Level 2 predictors in the model. We controlled for both mean negative emotions and depressive symptoms to align our analyses with Starr et al.’s (2020) analyses.

Making use of a recently proposed framework to study momentary emotion differentiation at the level of measurement occasions (Erbas et al., 2021), we additionally set out to explore within-person (i.e., in our case, daily) fluctuations in NED. More specifically, we calculated Erbas et al.’s (2021) novel momentary index of NED and analyzed whether the stress buffering effect of person-level NED translates to day-level NED (i.e., whether stress reactivity would be lower on days on which an individual’s NED is higher than usual). Moreover, as a first step toward elucidating a potential mechanism through which NED might exert a stress-buffering function, we additionally explored whether rumination about emotions would enhance negative responses to daily stress (i.e., whether daily rumination would act as a Level 1 moderator of the within-person link between daily stress and calmness in the evening) and whether lower daily NED would be associated with more daily rumination about emotions.

## Materials and Methods

We present the methodological details of our AA study by following the guidelines by Trull and Ebner-Priemer (2020).

### Study Design

The study consisted of an initial online survey and an AA phase across 21 days with two interval-based assessments per day (one morning survey and one evening survey). Participants chose a specific time schedule that best fit their waking hours (6 am/6 pm, 8 am/8 pm, or 10 am/10 pm). Data were collected using the software SoSci Survey (Leiner, 2020). Links to the daily surveys were sent via SMS, allowing participants to complete the surveys online using their own smartphone. Each link was valid for a certain time period (3 h for the morning survey, 6 h for the evening survey). During an initial online survey, participants completed a demographic questionnaire, COVID-19-related questions, and trait self-report measures. In the daily evening survey, participants rated their momentary mood and their experiences during the day (stress, positive and negative emotions, emotion regulation, worrying, and coping). In the morning survey, participants rated their momentary mood, the quality of their sleep from the previous night, and their expectations for the day. We selected a daily sampling schedule for experiences such as stress or emotions and a twice-per-day sampling schedule for momentary mood to fit the expected temporal variability of the constructs without overburdening participants.

### Procedure

Participants were recruited via mailing lists and social media platforms (Facebook, Twitter, and Instagram) and encouraged to inform family members, friends, and colleagues about the study. To be eligible, participants needed to be 15 years of age or older, to have access to a laptop, computer, or tablet in order to take part in the initial online survey, and to have access to a smartphone in order to participate in the AA phase. The AA phase spanned the same time period for each participant (April 13, 2020 through May 3, 2020). It began during the first complete pandemic lockdown due to COVID-19 in Germany (which had been established approximately 4 weeks prior to our assessment) when contact restrictions were implemented by the government. Toward the end of the AA phase, some protective measures were slowly lifted (e.g., the re-opening of small shops), and face mask policies were implemented.

All participants were informed about the study procedure via an information sheet on the registration webpage. They gave active consent to take part in the study via mouse click. Participants were reimbursed up to 60 EUR, partially contingent upon their compliance during the AA phase. The study procedure was approved by the institutional ethics committee of the psychology department at the University of Koblenz-Landau (258_2020).

### Participants

Seven hundred seventy-two individuals signed up to participate in the study. To achieve a more age-heterogeneous sample while at the same time complying with budgetary constraints on the total sample size of compensated participants, 311 individuals who had signed up and were between the ages of 20 and 29 (selected randomly) were not invited to participate. Four hundred sixty individuals (out of the 461 who were invited) began answering the initial online survey, of which 381 completed the entire initial online survey. Again, in the interest of achieving a more age-heterogeneous sample for the subsequent AA phase, out of the individuals between the ages of 20 and 25 who had filled out the online survey, 20 individuals per birth year (randomly selected from each birth year) were invited to participate in the AA phase of the study. This resulted in 52 participants aged 20–25 who were not invited to continue with the study and 329 participants who were invited to take part in the AA phase. Of these, 327 participants provided daily reports. Participants’ data were included in the statistical analyses if at least seven morning and seven evening surveys were available after checks for technical problems and careless responding (see the Compliance and section “Data Cleaning”). Data from five participants who did not fulfill this criterion were excluded. For the present analyses, the data of nine individuals with a negative ICC score (see section “Measures”) were excluded, leaving a final sample of 313 participants (74.1% women) between the ages of 15 and 82 (*M* = 30.1, *SD* = 14.9).

None of these 313 participants knowingly suffered from COVID-19 at the time of the initial online survey, but 55 participants (17.6%) reported cases of COVID-19 among their relatives or in their social environment. The prevalence of risk factors for severe COVID-19 in our sample was similar to estimates from a modeling study for Europe (Clark et al., 2020): 63.9% of participants indicated that they had no increased risk, 24% reported having one risk factor, and 12.1% reported having two or more risk factors. When asked for their level of concern regarding COVID-19 (ranging from 1 = *not at all* to 7 = *very much*), participants reported relatively low concerns about potential job loss (*M* = 2.72, *SD* = 1.87) and their individual financial situation (*M* = 3.10; *SD* = 1.88), moderate concerns about their own health (*M* = 3.45; *SD* = 1.68), and relatively high concerns about the health of their relatives (*M* = 5.44; *SD* = 1.51). These psychological reactions to the pandemic mirrored observations from representative surveys that were conducted during the same time period (Betsch et al., 2020).

### Measures

#### Within-Person (Daily) Measures

##### Daily stress

We measured daily subjective stress in the evening surveys with the item “How stressed did you feel today?” (Erbas et al., 2018). The response format was a visual slider that showed verbal anchors at each end (ranging from *not at all* to *very much*). The slider position selected by the participant was captured on a 101-point scale, which could be scaled as ranging, for instance, from 0 to 100. To avoid convergence issues in multilevel modeling due to the scaling of variables, we decided to scale the slider values as ranging from 0 to 1 in steps of 0.01.

##### Daily rumination about emotions

We measured daily rumination about emotions in the evening surveys with the item “I thought over and over again about my emotions” (Grommisch et al., 2020). Individuals indicated whether they had ruminated about their emotions during the day (0/*no*, 1/*yes*).

##### Momentary calmness

In both morning and evening surveys, we assessed momentary mood with an adapted short version of the Multidimensional Mood Questionnaire (Eid et al., 1999), which has previously been used in AA studies (e.g., Lischetzke et al., 2012). Two items tapped calmness [tense-relaxed, calm-uneasy (reverse-scored)]. Participants indicated how they felt at the moment using a bipolar visual slider scale that showed verbal anchors at each end (e.g., ranging from *tense* to *relaxed*). The slider position selected by the participant was captured on a 101-point scale, which was scaled as ranging from 0 to 1 in steps of 0.01. We calculated a mean score across the two items so that a higher score indicated a calmer mood. The reliability of the scale was estimated separately for the day level (within-person reliability) and the person level (between-person reliability) in accordance with Geldhof et al. (2014). Given that the scale consisted of two items, we calculated two-level alpha (because omega can only be calculated for at least three items). For evening (morning) assessments, within-person alpha was 0.77 (0.75), and between-person alpha was 0.96 (0.97).

##### Nightly sleep quality

We assessed subjective sleep quality in the morning surveys with three items [“How well did you sleep last night?” “How restlessly did you sleep last night?” (reverse-coded), “How easily did you fall asleep yesterday evening?”] that have been used in previous research (Åkerstedt et al., 2012; Könen et al., 2015). The response format was a 5-point Likert scale, ranging from 1 (e.g., *very poorly*) to 5 (e.g., *very well*). We calculated a mean score across the items so that a higher score indicated better sleep quality. To estimate the scale’s reliability, we calculated two-level omega (Geldhof et al., 2014). Within-person omega was 0.76, and between-person omega was 0.84.

##### Daily negative emotion differentiation

Each evening, participants indicated the intensity with which they had experienced eight negative emotions (anger, fear, disappointment, sadness, embarrassment/shame, regret, boredom, and loneliness) during the day. On the basis of an appraisal account of the affective space of discrete emotions (Scherer, 2005), we selected the items to represent negative emotions that differed on the appraisal dimension of coping potential/control (low: sadness, loneliness, fear; moderate: embarrassment/shame, disappointment, regret; high: boredom, anger). Participants rated the emotions on a visual slider scale ranging from not at all to very intense. The slider position selected by the participant was captured on a 101-point scale, which was scaled as ranging from 0 to 1 in steps of 0.01. If an emotion was not experienced at all during the day, participants were asked to set the slider to the far left. Because it may have been difficult for participants to indicate a value of exactly 0 on their smartphone touchscreen, we recoded all ratings ≤ 0.05 to 0 (cf. Koval et al., 2015). As an index of daily NED, we used the momentary index of emotion differentiation proposed by Erbas et al. (2021). More specifically, we applied the function calculate_ed from the R package emodiff described in Erbas et al. (2021) to calculate daily NED scores for each measurement occasion and each person. Resulting daily NED scores are more strongly negative when the level of momentary differentiation is low, and they approach zero when the level of differentiation is high (for details on the derivation of the momentary index from the classical between-person ICC index, see Erbas et al., 2021).

##### Daily mean of negative emotions

The daily negative emotion ratings were also used to compute an index of daily mean negative emotionality (by calculating the mean of all negative emotion items).

#### Between-Person (Trait) Measures

##### Depressive symptoms

To measure depressive symptoms, we used the nine-item depression module from the Patient Health Questionnaire (Spitzer et al., 1999; Gräfe et al., 2004), which is an instrument that is widely used to screen for mental disorders. In the initial online survey, participants rated the frequency of nine depressive symptoms during the past 2 weeks on a 4-point Likert scale ranging from 0 (*not at all*) to 3 (*almost every day*). We calculated a sum score across all the items, with higher scores indicating more depressive symptoms. Omega was 0.83.

##### Negative emotion differentiation

For each participant, we computed the ICC(3, *k*) measuring average consistency between negative emotions across measurement occasions (e.g., Erbas et al., 2014). Following previous recommendations (e.g., Kalokerinos et al., 2019; Erbas et al., 2021), negative ICC values were excluded from the analyses. This was the case for nine participants. Subsequently, ICC values were Fisher *Z*-transformed and reversed (multiplied by −1) so that higher values represented higher NED.

##### Mean level of negative emotions

The daily negative emotion ratings were also used to compute an index of mean negative emotionality experienced across the AA phase. For each participant, we calculated the mean of all negative emotion items across all measurement occasions.

### Data Cleaning

The 327 participants who completed the AA phase provided a total of 6,399 morning surveys and 6,519 evening surveys. Due to technical problems, for some of the assessments, the time window during which the surveys could be completed was longer than intended. Three morning surveys that had been completed after 1 p.m. as well as 34 evening surveys that had been completed after 4 a.m. were excluded from the analyses. One morning survey, which had erroneously been completed twice, was excluded from the analyses. Moreover, 11 morning and two evening surveys for which participants terminated their responding before they completed the first set of items (corresponding to sleep items in the morning and momentary mood items in the evening) were excluded from the analyses. To screen for careless responding, inconsistent responding across reverse-poled (momentary mood) items and response times were analyzed (Meade and Craig, 2012). Eighty-one morning and 100 evening surveys were excluded due to inconsistent responding, and three morning and 282 evening surveys were excluded due to extremely short response times^1^. Subsequently, we excluded data from five participants who completed fewer than seven morning and seven evening surveys, leaving a sample of 6,084 evening surveys and 6,263 morning surveys nested in 322 participants.

### Final Sample and Compliance

For the present analyses, the data of nine participants whose ICC values were negative were excluded (see section “Measures”). This resulted in a sample of 5,912 evening surveys and 6,095 morning surveys nested in 313 participants. On average, participants provided 18.89 (out of 21 possible) evening surveys (*SD* = 2.83, *Min* = 7, *Max* = 21) and 19.47 (out of 21 possible) morning surveys (*SD* = 1.96, *Min* = 11, *Max* = 21). For the mediation analyses in the present paper, we included evening surveys from Day 1 through Day 20 (*n* = 5,645 surveys) and merged them with the morning mood ratings from the same day (i.e., from Day 1 to Day 20; *n* = 5,303 surveys) as well as with the sleep quality ratings collected the next morning (i.e., from Day 2 to Day 21; *n* = 5,302 surveys). The reason for excluding the data from Day 21 was that the sleep-quality ratings referring to this day were missing by design. Hence, the analyses in the present paper were based on a total of 5,645 days nested in 313 individuals.

### Sample Size Considerations

According to a simulation study on the power to detect a cross-level interaction in multilevel modeling (Mathieu et al., 2012), a combination of 115 Level 2 units and 18 Level 1 units per Level 2 unit yielded a power of larger than 0.80 to detect a medium-sized cross-level interaction effect. Given that the size of our sample (313 persons and 18.89 evening assessments per person, on average) met or exceeded these sample sizes, we deemed our data set large enough to test our central hypothesis that NED would be found to moderate the within-person indirect effect of daily stress on daily sleep quality using a moderated multilevel (1-1-1) mediation model.

### Analytic Strategy

We applied a multilevel structural equation modeling (MSEM) approach using Bayesian estimation (Asparouhov and Muthén, 2019) with default uninformative priors in Mplus Version 8.5 (Muthén and Muthén, 1998-2020). The advantages of using Bayesian estimation (as a pragmatic approach) for multilevel mediation models with multiple random effects are that latent centering of observed time-varying variables can be applied (Asparouhov and Muthén, 2019), standardized parameter estimates (and estimates of level-specific *R*^2^) can be obtained, and a non-symmetric Bayesian credibility interval, which does not assume normality, can be used to evaluate the significance of the estimated within-person indirect effect (Muthén, 2010). To evaluate convergence, we inspected whether the parameter estimates and the potential scale reduction (PSR) values (obtained via the Mplus TECH8 output) changed when we increased the number of iterations to 10,000 (e.g., Zyphur and Oswald, 2015). With the latent centering method in MSEM, the observed daily variables *X*_*ti*_, *M*_*ti*_, and *Y*_*ti*_ (where *t* represents days and *i* represents persons) are decomposed into a within-person part (*X*_W_, *M*_W_, and *Y*_W_) and a between-person part (*X*_B_, *M*_B_, and *Y*_B_).

To test the within-person effects of daily stress (*X*_W_) on calmness in the evening (*M*_W_) and that night’s sleep quality (*Y*_W_), we specified a lower level (1-1-1) mediation model (Model 1; see the Level 1 part of Figure 1) with random slopes for all Level 1 path coefficients (Preacher et al., 2010). Two additional Level 1 variables were included as control variables: To rule out the possibility that within-person relationships between *X*_W_, *M*_W_, and *Y*_W_ were simply due to shared time trends in these variables across the study period, time (centered at Day 11 and coded so that the total study time represented a time unit of 1) was included as a predictor of *M*_W_ and *Y*_W_ (Note that for simplicity, time is not depicted as a predictor in Figure 1). Calmness in the morning was included as a Level 1 (person-mean-centered) predictor of calmness in the evening so that path *a*_*i*_ represented the within-person relation between daily stress and within-day change in mood. At the between-person level (Level 2), we allowed the between-person slopes *a*_*i*_, *b*_*i*_, and *c*_*i*_’ and the between-person intercepts to correlate freely (Preacher et al., 2016). The average within-person indirect effect is defined as *E*(*a*_*i*_*b*_*i*_) = *ab* + σ_*a*_*i*_,*b*_*i*__, where *a* is the mean of the random slopes *a*_*i*_, *b* is the mean of the random slopes *b*_*i*_, and σ_*a_i_,b_i_*_ is the covariance between the random slopes *a*_*i*_ and *b*_*i*_ (Bauer et al., 2006). We expressed the average within-person indirect effect as a model constraint in Mplus and evaluated it on the basis of the estimated (non-symmetric) 95% Bayesian credibility interval (which uses the 2.5th and 97.5th percentiles of the posterior distribution, thus allowing for skewness). Note that establishing mediation does not require the total effect of *X*_W_ on *Y*_W_ to be significant (MacKinnon et al., 2000). One reason for this is that the statistical test of the total effect can have less power than the test of the indirect effect (Hayes, 2009; MacKinnon and Fairchild, 2009).

In the next step, to test whether the within-person relation between daily stress (*X*_W_) and evening mood (*M*_W_) varied as a function of NED, we extended the model to a first-stage multilevel conditional process model (Hayes and Rockwood, 2020). That is, NED was added as a Level 2 predictor of the random slope term *a*_*i*_ (Model 2; see Figure 1). Note that NED was also added as a predictor of the random intercept term for calmness in the evening because main effects always have to be included when testing for a moderator effect. To enhance the interpretation of the model estimates, NED was grand-mean centered. To probe the cross-level interaction, we estimated the conditional effect of *X*_W_ on *M*_W_ at high (*M* + 1 *SD*) and low (*M* – 1 *SD*) values of NED as well as the conditional indirect effect of *X*_W_ on *Y*_W_ through *M*_W_ at those values of NED.

To test whether the hypothesized moderator effect of NED held when we controlled for between-person differences in mean negative emotions and depressive symptoms, these variables were added as grand-mean-centered predictors of the random slope term *a*_*i*_ (and as predictors of the random intercept term for calmness in the evening) at Level 2 (Model 3).

In our supplementary analyses, we explored (a) whether evidence for a stress buffering of NED could be found when daily NED (instead of person-level NED) was analyzed (i.e., whether daily NED would moderate the within-person link between daily stress and calmness in the evening), and (b) whether this effect held when daily mean negative emotions were controlled. To analyze (a), we specified a two-level model in which daily stress, daily NED, and their interaction predicted calmness in the evening at Level 1. Following Enders and Tofighi’s (2007) recommendations, we centered both the continuous variable (daily NED) and the dichotomous variable (rumination) at the person mean and subsequently computed the interaction term. Again, calmness in the morning and time were included as Level 1 control variables. To analyze (b), we added person-mean centered daily mean negative emotions as a Level 1 predictor. Moreover, to examine a potential mechanism through which NED might exert a stress buffering effect, we explored (c) whether daily rumination about emotions would moderate the within-person link between daily stress and calmness in the evening. We computed the Level 1 interaction term between daily stress and daily rumination about emotions and set up the model in the same way as described for the model involving the interaction between daily stress and daily NED. Finally, we explored (d) whether lower daily NED would predict a higher probability of ruminating about emotions, and (e) whether this relation would hold when daily mean negative emotions were controlled. To do so, we added daily NED and daily mean negative emotions as Level 1 predictors of daily rumination to model (c).

## Results

### Descriptive Statistics

Correlations and descriptive statistics for the day-level and person-level variables are provided in Tables 1, 2.

**TABLE 1:** Within- and between-person correlations and descriptive statistics for daily variables.

Variable	1	2	3	4	5	6
1. Calmness in the morning	–	−0.61***	0.87***	0.53***	−0.22***	−0.53***
2. Daily stress	−0.17***	–	−0.62***	−0.44***	0.27***	0.57***
3. Calmness in the evening	0.13***	−0.32***	–	0.52***	−0.25***	−0.63***
4. Nightly sleep quality	–0.01	–0.02	0.08***	–	−0.19**	−0.42***
5. Daily rumination^1^	−0.05**	0.12***	−0.13***	0.02	–	0.41***
6. Daily mean neg. emotions	−0.11***	0.35***	−0.36***	−0.04**	0.22***	–
7. Daily NED^2^	0.02	−0.15***	0.17***	0.03*	−0.13***	−0.46***
*M*	0.67	0.41	0.70	3.75	0.38	0.22
*SD*_within_	0.15	0.23	0.16	0.74	–	0.12
*SD*_between_	0.16	0.14	0.14	0.47	–	0.12
ICC	0.53	0.28	0.43	0.29	0.49	0.52
Range	0–1	0–1	0–1	1–5	0–1	0–1

N_Level 1_ = 5,645 days, N_Level 2_ = 313 persons. Within-person correlations are presented below the diagonal, and between-person correlations are presented above the diagonal. M, grand mean (i.e., the mean across days and persons); SD_within_, within-person standard deviation; SD_between_, between-person standard deviation; ICC, intraclass correlation coefficient. ^1^For the binary variable daily rumination (0/no, 1/yes), the mean represents the average proportion of days on which individuals ruminated about their emotions, and the intraclass correlation was estimated using Goldstein et al.’s (2002) method D. ^2^By definition, daily NED (i.e., the momentary differentiation index by Erbas et al., 2021) is a within-person variable. Therefore, the ICC is 0, and only within-person correlations are depicted. For information on the person-level index of NED, see Table 2. Descriptive statistics for daily NED were M = −2.43, SD = 4.27, Range: −58.29–0. *p < 0.05; **p < 0.01; ***p < 0.001.

**TABLE 2:** Between-person correlations and descriptive statistics for person-level variables.

Variable	1	2
*Trait variables*		
1. NED	–	
2. Depressive symptoms	–0.09	–
*Daily variables (between-person part)*		
3. Calmness in the morning	0.13*	−0.45***
4. Daily stress	–0.11	0.39***
5. Calmness in the evening	0.16**	−0.49***
6. Nightly sleep quality	0.09	−0.51***
7. Daily rumination	–0.08	0.34***
8. Daily mean negative emotions	−0.20***	0.58***
*M*	0.36	7.57
*SD*	0.19	4.85
Range	0.08–1	0–27

N = 313 persons. To aid in interpretability of the mean for NED, we report the descriptive statistics for the raw scores (i.e., prior to Fisher’s Z-transformation), reverse scored. *p < 0.05; **p < 0.01; *** p < 0.001.

### Multilevel (1-1-1) Mediation Model

The fixed effects of the multilevel (1-1-1) mediation model (including time and calmness in the morning as Level 1 control variables) are displayed in Table 3 (Model 1). The fixed effects of time represent the average within-person trajectories in evening mood and sleep quality across the study phase. On average, sleep quality significantly increased by 0.111 points (on a 1–5 scale) across the total duration of the study of 3 weeks, which might be indicative of a small overall effect of participants’ adaptation to the (first-ever) pandemic lockdown in Germany.

**TABLE 3:** Estimates for (moderated) multilevel (1-1-1) mediation models.

	Model 1					Model 2				
Coefficients	Est.	Post. *SD*	One-tailed *p*	95% CI	Stand. est.	Est.	Post. *SD*	One-tailed *p*	95% CI	Stand. est.
*Fixed effects*										
Time → *M*_W_	0.015	0.008	0.029	(−0.001, 0.032)	0.026	0.015	0.009	0.048	(−0.003, 0.033)	0.026
Time → *Y*_W_	0.111	0.043	0.005	(0.021, 0.194)	0.042	0.111	0.042	0.006	(0.025, 0.194)	0.041
CalmMor_W_ → *M*_W_	0.067	0.017	<0.001	(0.035, 0.103)	0.061	0.070	0.018	<0.001	(0.033, 0.106)	0.062
***X*_W_ → *M*_W_ (*a*)**	**−−0.222**	**0.014**	**<0.001**	**(−0.250, −0.195)**	**−−0.301**	**−−0.225**	**0.013**	**<0.001**	**(−0.251, −0.197)**	**−−0.303**
***M*_W_ → *Y*_W_ (*b*)**	**0.412**	**0.081**	**<0.001**	**(0.254, 0.577)**	**0.086**	**0.372**	**0.085**	**<0.001**	**(0.215, 0.546)**	**0.078**
*X*_W_ → *Y*_W_ (*c′*)	0.011	0.051	0.427	(−0.100, 0.095)	0.002	–0.011	0.058	0.417	(−0.142, 0.091)	–0.004
**NED → *a*_*i*_**						**0.107**	**0.041**	**0.007**	**(0.021, 0.183)**	**0.161**
NED → *M*_B_						0.039	0.019	0.022	(0.001, 0.077)	0.063
**Indirect effect [*E*(*a_*i*_b_*i*_*)]**	**−−0.063**			**(−0.109, −0.014)**		**−−0.050**			**(−0.097, −0.004)**	
Total effect [*E*(*a_*i*_b_*i*_*) + *c′*]	–0.054			(−0.155, 0.033)		–0.063			(−0.179, 0.036)	
*R* ^2^ *at Level 1*										
*R*^2^ (*M*_W_)	0.172					0.173				
*R*^2^ (*Y*_W_)	0.055					0.056				
*R* ^2^ *at Level 2*										
*R*^2^ (*a*_*i*_)						0.026				
*R*^2^ (*M*_B_)						0.004				

*Focal effects of the (moderated) mediation model are bolded. Index W (B) indicates the within- (between-) person part of time-varying variables. The covariance (correlation) between the random slopes a_i_ and b_i_ was 0.029 (0.269) in Model 1 and 0.033 (0.303) in Model 2. X, Daily stress; M, calmness in the evening; Y, nightly sleep quality; CalmMor, calmness in the morning; Est., estimate; Stand. Est., standardized estimate; Post. SD, posterior standard deviation; One-tailed p, Bayesian one-tailed p-value; CI, Bayesian credibility interval.*

The results supported Hypothesis 1 on the within-person indirect effect of daily stress (*X*_W_) on daily sleep quality (*Y*_W_) through calmness in the evening (*M*_W_), *E*(*a_*i*_b_*i*_*) = −0.063 (see Model 1, Table 3). As expected, higher daily stress was related to less calmness in the evening within persons (*a* = −0.222), and this effect was moderate in size. Less calmness in the evening in turn was related to worse sleep quality within persons (*b* = 0.412), and this effect was small in size.

Of note, individuals differed significantly in the within-person relations (as indicated by variance estimates for the random slope terms whose 95% credibility intervals did not include 0). To examine the patterns of individual differences in within-person relations in more detail, we calculated the percentage of slopes < 0 and the 95% predictive interval for paths *a*_*i*_ and *b*_*i*_ (Hox et al., 2018). Assuming a normal distribution of random slopes, the percentage of slopes < 0 indicates the proportion of regression slopes that is estimated to be negative, and the 95% predictive interval indicates the range of values between which 95% of the person-specific regression slopes are estimated to lie. For path *a*_*i*_, the percentage of slopes < 0 was 92%, and the 95% predictive interval was (−0.526, 0.082) [corresponding to standardized estimates of (−0.734, 0.114)]. For path *b*_*i*_, the percentage of slopes < 0 was 28%, and the 95% predictive interval was (−1.008, 1.832) [corresponding to standardized estimates of (−0.223, 0.407)]. That is, for our focal path *a*_*i*_, whose random slopes represent individual differences in stress reactivity, this means that a negative link between daily stress and calmness in the evening was estimated for the large majority of individuals—however, the size of this relationship differed greatly across individuals.

### Moderated Multilevel (1-1-1) Mediation Models (First-Stage Conditional Process Models)

**FIGURE 2:**
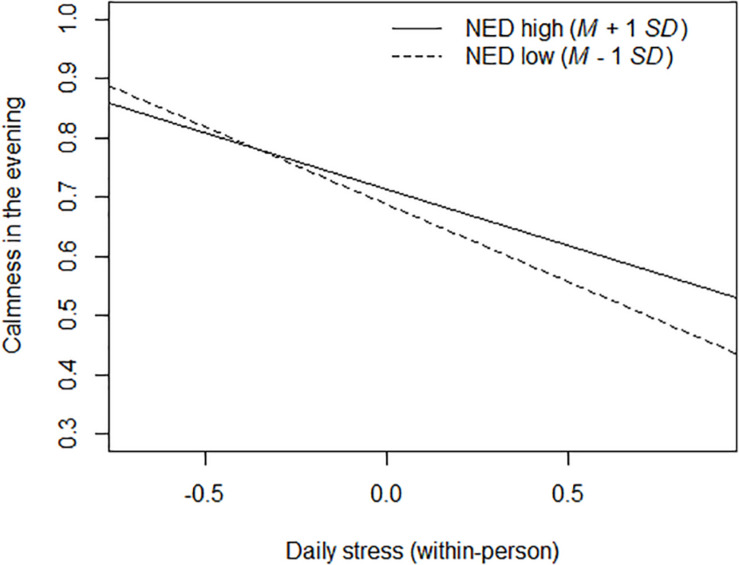
Simple slopes for the moderator effect of NED on the within-person relation between daily stress and calmness in the evening.

Next, we entered NED as a Level 2 predictor to the model. The fixed effects of the moderated multilevel (1-1-1) mediation model are displayed in Table 3 (Model 2). NED predicted higher calmness in the evening (main effect of NED on the varying intercepts), and this corresponded to a small effect size (see row NED → *M*_B_ in Table 3). Supporting Hypothesis 2, NED positively predicted the varying random slopes for the effect of daily stress on calmness in the evening (see row NED → *a*_*i*_). The within-person relation between daily stress and less calmness in the evening was stronger at low (*M* – 1 *SD*) NED, simple slope estimate = −0.261, 95% CI (−0.297, −0.224), than at high (*M* + 1 *SD*) NED, simple slope estimate = −0.189, 95% CI (−0.226, −0.151). Figure 2 illustrates this cross-level interaction. Additionally, we estimated the conditional within-person indirect effect of *X*_W_ on *Y*_W_ through *M*_W_ at low (*M* – 1 *SD*) and high (*M* + 1 *SD*) values of NED. This was done by centering NED at these values of interest and re-running the model (Hayes and Rockwood, 2020). The estimated indirect effect then represented the conditional indirect effect when NED was equal to that specific value of interest. For individuals with low NED, the estimated within-person indirect effect was significant, *E*(*a_*i*_b_*i*_*) = −0.061, 95% CI (−0.116, −0.014). For individuals with high NED, the estimated within-person indirect effect was non-significant, *E*(*a_*i*_b_*i*_*) = −0.037, 95% CI (−0.082, 0.007).

Finally, we controlled for mean negative emotions and depressive symptoms at Level 2 (Model 3). The fixed effects results for this model can be found in Table 4. Both mean negative emotions and depressive symptoms predicted the intercept for calmness in the evening (see rows NegEmo → *M*_B_ and Depr → *M*_B_ in Table 4), whereas the main effect of NED on calmness in the evening (row NED → *M*_B_) was no longer different from zero. The cross-level interaction of NED and daily stress on calmness in the evening was retained (see row NED → *a*_*i*_). Neither mean negative emotions nor depressive symptoms moderated the within-person relation between daily stress and calmness in the evening (see rows NegEmo → *a*_*i*_ and Depr → *a*_*i*_)^2^.

**TABLE 4:** Estimates for moderated multilevel (1-1-1) mediation model including level 2 control variables (Model 3).

Coefficients	Estimate	Post. *SD*	One-tailed *p*	95% CI	Stand. estimate
*Fixed effects*					
Time → *M*_W_	0.015	0.008	0.034	(−0.002, 0.032)	0.027
Time → *Y*_W_	0.117	0.040	0.002	(0.045, 0.197)	0.043
CalmMor_W_ → *M*_W_	0.072	0.018	<0.001	(0.038, 0.113)	0.063
***X*_W_ → *M*_W_ (*a*)**	**−0.221**	**0.014**	**<0.001**	**(−0.251, −0.194)**	**−0.299**
***M*_W_ → *Y*_W_ (*b*)**	**0.402**	**0.085**	**<0.001**	**(0.223, 0.558)**	**0.082**
*X*_W_ → *Y*_W_ (*c′*)	0.006	0.056	0.470	(−0.096, 0.114)	−0.004
**NED → *a*_*i*_**	**0.109**	**0.042**	**0.002**	**(0.028, 0.189)**	**0.160**
NegEmo → *a*_*i*_	0.077	0.143	0.286	(−0.170, 0.411)	0.045
Depr → *a*_*i*_	−0.005	0.004	0.120	(−0.012, 0.002)	−0.107
NED → *M*_B_	0.019	0.018	0.130	(−0.015, 0.054)	0.037
NegEmo → *M*_B_	−0.363	0.061	<0.001	(−0.487, −0.246)	−0.266
Depr → *M*_B_	−0.003	0.001	0.014	(−0.006, 0.000)	−0.089
**Indirect effect [*E*(*a_*i*_b_*i*_*)]**	**−0.065**			**(−0.114, −0.007)**	
Total effect [*E*(*a_*i*_b_*i*_*) + *c′*]	−0.056			(−0.164, 0.041)	
*R* ^2^ *at Level 1*					
*R*^2^ (*M*_W_)	0.172				
*R*^2^ (*Y*_W_)	0.060				
*R* ^2^ *at Level 2*					
*R*^2^ (*a*_*i*_)	0.049				
*R*^2^ (*M*_B_)	0.083				

*Focal effects of the moderated mediation model are bolded. Index W (B) indicates the within- (between-) person part of time-varying variables. The covariance (correlation) between the random slopes a_i_ and b_i_ was 0.024 (0.215). X, daily stress; M, calmness in the evening; Y, nightly sleep quality; CalmMor, calmness in the morning; NegEmo, mean negative emotions; Depr, depressive symptoms; Post. SD, posterior standard deviation; One-tailed p, Bayesian one-tailed p-value; CI, Bayesian credibility interval.*

### Supplementary Analyses

In our supplementary analyses, we first explored (a) whether the stress buffering effect that we found for person-level NED translates to day-level NED (i.e., whether stress reactivity would be lower on days on which an individual’s momentary differentiation is higher than usual). In a two-level model predicting calmness in the evening by daily stress and daily NED and their interaction (controlling for calmness in the morning and time), the Level 1 interaction term was significant [estimate = 0.013, 95% CI (−0.007, 0.022)]. As expected, on days with higher NED, the stress-calmness link was less negative [simple slope estimate = −0.143, 95% CI (−0.181, −0.109)] than on days with lower NED [simple slope estimate = −0.258, 95% CI (−0.301, −0.220)]. However, when (b) daily negative mean emotions was added to the model as a Level 1 predictor, the Level 1 interaction term was no longer significant [estimate = 0.002, 95% CI (−0.004, 0.008)].

Additionally, we explored daily rumination as a potential mechanism through which NED might exert its stress-buffering effect. In model (c), we analyzed whether daily rumination about emotions moderated the within-person relation between daily stress and calmness in the evening (controlling for calmness in the morning and time). A two-level model revealed a significant Level 1 interaction between daily stress and daily rumination [estimate = −0.052, 95% CI (−0.109, −0.003)]. On the days on which individuals ruminated, the negative stress-calmness link was stronger [estimate = −0.242, 95% CI (−0.277, −0.200)] than on days on which individuals did not ruminate about their emotions [estimate = −0.191, 95% CI (−0.225, −0.160)]. In the next step (model d), we added daily NED to the model as a Level 1 predictor of daily rumination. The regression coefficient for daily NED was significant [estimate = −0.008, 95% CI (−0.012, −0.004)]. That is, on days with lower NED, the probability to ruminate was higher. However, when we added daily mean negative emotions as an additional Level 1 predictor of daily rumination (model e), this effect vanished [estimate = −0.002, 95% CI (−0.005, −0.002)].

## Discussion

With the current AA study, we aimed to investigate the indirect within-person effect of perceived daily stress on subjective sleep quality through calmness in the evening in a community sample of adults during times of stress (the first pandemic lockdown in 2020). Our main moderator hypothesis represented a conceptual replication and extension of Starr et al.’s (2017, 2020) findings on the role of NED in buffering daily stress reactivity. As expected, higher daily stress was related to within-day change in calmness from morning to evening, resulting in less calmness in the evening within persons. Less calmness in the evening, in turn, was related to poorer nightly sleep quality within persons. Supporting our main hypothesis, NED moderated the within-person relation between daily stress and calmness in the evening, with lower NED predicting a stronger negative link between daily stress and calmness in the evening. This also meant that the indirect within-person effect of daily stress on sleep quality through calmness in the evening was found to be conditional on an individual’s standing on NED. For low differentiators, daily stress was negatively linked to sleep quality through calmness in the evening, whereas for high differentiators, this within-person indirect effect was non-significant.

Ambulatory assessment studies on within-person processes linking day-to-day fluctuations in stress to affective states prior to sleep and to sleep quality that night are still scarce (cf. Tousignant et al., 2019). Our result that the within-person indirect effect of daily stress on sleep quality through calmness in the evening was, on average, negative is consistent with previous research that found that cognitive and somatic arousal at bedtime mediated the link between daily stress and subjective sleep quality (Morin et al., 2003; Winzeler et al., 2014; Tousignant et al., 2019). Despite variation with respect to the concrete operationalization of calmness/tense arousal (ratings of mood adjectives in our study vs. ratings of statements describing cognitive processes and felt somatic states in the cited studies), the time point that was referred to (evening vs. bedtime), and the type of assessment that was used (e.g., momentary mood ratings collected in the evening or before going to sleep vs. retrospective judgments collected in the morning in Tousignant et al.’s study), the results converged in showing that more negative affective reactions to daily stress predicted impaired sleep quality within persons. Calmness in the evening could be important because falling asleep requires the inhibition of multiple arousal systems (Szymusiak and McGinty, 2008), and the ease with which a person falls asleep is a crucial aspect of sleep quality (Åkerstedt et al., 1994, 2012). There is some empirical evidence suggesting that the relation between nightly sleep and daily affect may be bidirectional (Konjarski et al., 2018), potentially resulting in a vicious circle of tense arousal and disturbed sleep (Garde et al., 2011). In our study, we decided to analyze nightly sleep quality as an outcome variable because our focus was on (individual differences in) daily stress reactivity and its consequences. However, we controlled for daily “baseline levels” of calmness in our models (by entering calmness in the morning as an additional predictor of calmness in the evening) to reduce the possibility that inverse effects of sleep quality on the next day’s tense arousal would bias our models’ within-person estimates.

At the person level, we found that NED had a small association with higher calmness across the study period of 3 weeks. When we controlled for mean negative emotions (and depressive symptoms), this “main effect” of NED on average calmness vanished. This finding is in line with results from Dejonckheere et al. (2019), who showed that small relations between NED and well-being indicators became non-significant when mean affect was controlled for. Unique explanatory power of NED over and above reliable trait-like measures of affective functioning would be expected for outcome variables in which a considerable amount of variance is due to more complex temporal dynamics (e.g., Dejonckheere et al., 2019). Thus, the disappearing predictive utility of NED when mean negative emotions were controlled might be particularly informative about the outcome measure: Average calmness across 3 weeks during an uncertain time (a pandemic lockdown) can be considered as an indicator of individuals’ dispositional affective functioning. Moreover, we found that NED was unrelated to depressive symptoms and average sleep quality. Previous research has revealed small to moderate negative correlations between NED and depressive symptoms in healthy populations (Erbas et al., 2014; Starr et al., 2017, 2020; Dejonckheere et al., 2019). However, this link may also be mainly due to the variance that both NED and depressive symptoms share with mean negative emotions (Dejonckheere et al., 2019). Taken together, our non-significant “main effects” of NED at the person level underscore the need to scrutinize within-person regulatory processes more closely because “it is possible that unique associations between affect dynamics and psychological well-being exist, but that current research practices leave it undisclosed” (Dejonckheere et al., 2019, p. 486).

The results of our moderated multilevel (1-1-1) mediation analysis conceptually replicated and extended Starr et al.’s (2017, 2020) findings on the stress-buffering effect of NED. In a community sample of adults, and using calm mood (instead of depressive mood) as an indicator of stress reactivity, we found evidence for the expected moderating effect of NED on the within-person relation between daily stress and calmness. Importantly, the stress buffering effect of NED was not accounted for by individual differences in mean negative emotions and depressive symptoms. That is, our results provide additional support for Starr et al.’s (2020) diathesis-stress model of NED, and hence, for NED as a protective factor that helps to explain why some individuals remain more resilient during times of stress than others. Moreover, our finding that the indirect within-person effect of daily stress on nightly sleep quality via calmness in the evening was negative for low differentiators and not significantly different from zero for high differentiators hints at within-person processes through which NED might confer health-related benefits during times of stress. Finally, it is important to note that the cross-level interaction between NED and daily stress could also be interpreted to demonstrate that the predictive power of NED is limited to specific situational conditions: In line with theoretical reasoning (Kashdan et al., 2015; O’Toole et al., 2020) and previous empirical evidence (Ottenstein and Lischetzke, 2020), high (vs. low) NED was most beneficial on stressful days that presented a challenge to a person’s well-being—that is, when the need for regulation was greatest.

In our supplemental analyses, we additionally scrutinized whether the stress buffering effect of person-level NED could also be found for within-person fluctuations in NED. When applying the recently proposed momentary index of emotion differentiation (Erbas et al., 2021) to our data, we found that daily NED moderated the within-person stress-calmness link, and the form of this Level 1 interaction was similar to the form of the cross-level interaction. However, in contrast to the person level, where the stress buffering effect of NED held beyond mean negative emotions (and depressive symptoms), the moderator effect of daily NED was not significant after controlling for daily mean negative emotions. One reason for this might be that the shared variance between NED and mean negative emotions was smaller at the between-person level (*r* = −0.20) than at the within-person level (*r* = −0.46). A moderate to high negative correlation between the momentary index of NED and the mean negative emotion scores at each occasion is expected to occur if the mean emotion scores are right-skewed (see Erbas et al., 2021), which is typical for negative emotions and was also the case in our study. Another aim of our supplementary analyses was to explore whether a reduced tendency to ruminate about emotions might represent a potential mechanism through which NED exerts its stress-buffering effect. In line with previous findings on the deleterious effect of daily rumination on affect (e.g., Puterman et al., 2010; Catalino et al., 2017), the within-person link between daily stress and calmness in the evening was more negative on days on which individuals ruminated about their emotions. Lower daily NED predicted a higher tendency to ruminate about emotions, thus providing support for a strategy selection effect of NED (Kalokerinos et al., 2019). However, the association with daily rumination was not unique for daily NED because the predictive power of NED disappeared when we controlled for daily mean negative emotions. Given the novelty of the momentary index of emotion differentiation, more research is needed on the conditions under which it shows predictive utility beyond mean affect. This might include assessment-related aspects such as the selection of emotions (which differ in the frequency and intensity with which they are experienced in daily life), design-related aspects such as the degree of variability in situational context individuals are in during the study phase, or substantive aspects such as outcome variables that refer to different points in the affect regulation process.

### Limitations

Our measure of daily stress was a retrospective measure collected in the evening. Retrospective end-of-day measures have been shown to converge strongly with aggregated momentary ratings within persons (Neubauer et al., 2020). Nonetheless, participants’ daily stress ratings might have been affected by their momentary mood when completing the end-of-day assessment. To control for potential effects of day-to-day fluctuations in mood on the perception of daily stress, we assessed calmness in the morning and included it as a control variable in our analyses. Still, we cannot fully rule out an effect of momentary mood in the evening on the daily stress rating. Therefore, more research is needed to scrutinize whether an alternative way to measure daily stress (for instance, by assessing momentary stress multiple times during the day instead of retrospectively in the evening) would yield similar findings.

Although our study is one of only a few studies to date that have examined the predictive utility of NED with respect to individual differences in within-person regulatory processes, it remains unclear whether the stress-buffering effect of NED translates into longer term resilience against adversity. Future research could use multiple intensive assessment phases separated by longer time intervals (i.e., measurement burst designs) to study both short- and longer-term outcomes.

Despite rates of individual COVID-19-related risk factors that were comparable to those in the general population in Europe (Clark et al., 2020) and psychological reactions to the pandemic that were similar to those in the general German population during the first lockdown in 2020 (Betsch et al., 2020), our community sample was not representative in other respects: women between the ages of 20 and 29 were overrepresented. Therefore, the results might not be generalizable beyond a female, young adult population.

### Conclusion

The present study adds to the growing literature on the role of individual differences in NED in within-person affect regulation processes. Our findings support the notion that higher NED buffers daily stress reactivity and thereby attenuates the negative indirect effect of daily stress on nightly sleep quality. The unique predictive utility of NED (beyond mean negative emotions and depression) was found for the prediction of individual differences in these within-person regulatory processes but not for the prediction of individual differences in mean levels of well-being indicators (e.g., average calm mood or average sleep quality). This discrepancy underscores the need for more process-oriented research to investigate the specific benefits that the ability to differentiate discrete negative emotions might confer.

## Data Availability Statement

The raw data supporting the conclusions of this article will be made available by the authors, without undue reservation.

## Ethics Statement

The studies involving human participants were reviewed and approved by the Institutional Ethics Committee of the Psychology Department at the University of Koblenz-Landau. Written informed consent from the participants’ legal guardian/next of kin was not required to participate in this study in accordance with the National Legislation and the Institutional Requirements.

## Author Contributions

LS and TL organized the database. TL performed the statistical analyses and wrote the first draft of the manuscript. All authors contributed to conception and design of the study, manuscript revision, read, and approved the submitted version.

## Conflict of Interest

The authors declare that the research was conducted in the absence of any commercial or financial relationships that could be construed as a potential conflict of interest.

## Publisher’s Note

All claims expressed in this article are solely those of the authors and do not necessarily represent those of their affiliated organizations, or those of the publisher, the editors and the reviewers. Any product that may be evaluated in this article, or claim that may be made by its manufacturer, is not guaranteed or endorsed by the publisher.

## Footnotes

1 The response time cutoff values were determined by conducting a pilot AA study (that contained the same items) in which research assistants were instructed to complete the morning and evening surveys as quickly as possible without switching to careless responding. In this pilot study, the fastest response time for a morning survey was 1.17 s/item, and the fastest response time for an evening survey was 1.56 s/item (We attributed the shorter response time for the morning survey to the fact that items in the evening survey included more text, on average, than items in the morning survey). In the main study, surveys with a response time below these cutoff values were excluded from the analyses.

2 To explore whether the within-person relation between calmness in the evening and sleep quality (path *b*_*i*_) varied as a function of NED, we additionally ran a first- and second-stage multilevel conditional process model in which NED predicted the random slope terms *a*_*i*_ and *b*_*i*_ (and the two random intercept terms for evening mood and sleep quality). NED did not moderate the random slope term *b*_*i*_ (estimate = −0.267, posterior *SD* = 0.247, one-tailed Bayesian *p* = 0.138, 95% CI (−0.752, 0.210)], nor did NED predict the intercept term for sleep quality (estimate = 0.067, posterior *SD* = 0.080, one-tailed Bayesian *p* = 0.200, 95% CI (−0.090, 0.223)].

## References

[B1] Åkerstedt T. Hume K. Minors D. Waterhouse J. (1994). The subjective meaning of good sleep, an intraindividual approach using the Karolinska Sleep Diary. *Percept. Motor Skills* 79 287–296. 10.2466/pms.1994.79.1.287 7991323

[B2] Åkerstedt T. Orsini N. Petersen H. Axelsson J. Lekander M. Kecklund G. (2012). Predicting sleep quality from stress and prior sleep—a study of day-to-day covariation across six weeks. *Sleep Med.* 13 674–679. 10.1016/j.sleep.2011.12.013 22621983

[B3] Asparouhov T. Muthén B. (2019). Latent variable centering of predictors and mediators in multilevel and time-series models. *Struct. Equ. Modeling* 26 119–142. 10.1080/10705511.2018.1511375

[B4] Barrett L. F. Gross J. Christensen T. C. Benvenuto M. (2001). Knowing what you’re feeling and knowing what to do about it: mapping the relation between emotion differentiation and emotion regulation. *Cogn. Emot.* 15 713–724. 10.1080/02699930143000239

[B5] Bauer D. J. Preacher K. J. Gil K. M. (2006). Conceptualizing and testing random indirect effects and moderated mediation in multilevel models: new procedures and recommendations. *Psychol. Methods* 11 142–163. 10.1037/1082-989X.11.2.142 16784335

[B6] Betsch C. Korn L. Felgendreff L. Eitze S. Schmid P. Sprengholz P. (2020). *German COVID-19 Snapshot Monitoring (COSMO) - Welle 7 (14.04.2020). PsychArchives* [Preprint]. Available online at: 10.23668/PSYCHARCHIVES.2875 (accessed September 1, 2020).

[B7] Boden M. T. Thompson R. J. Dizén M. Berenbaum H. Baker J. P. (2013). Are emotional clarity and emotion differentiation related? *Cogn. Emot.* 27 961–978. 10.1080/02699931.2012.751899 23237455

[B8] Catalino L. I. Arenander J. Epel E. Puterman E. (2017). Trait acceptance predicts fewer daily negative emotions through less stressor-related rumination. *Emotion* 17 1181–1186. 10.1037/emo0000279 28406676 PMC5640455

[B9] Clark A. Jit M. Warren-Gash C. Guthrie B. Wang H. H. X. Mercer S. W. (2020). Global, regional, and national estimates of the population at increased risk of severe COVID-19 due to underlying health conditions in 2020: a modelling study. *Lancet Glob. Health* 8 e1003–e1017. 10.1016/S2214-109X(20)30264-3 32553130 PMC7295519

[B10] Dejonckheere E. Mestdagh M. Houben M. Rutten I. Sels L. Kuppens P. (2019). Complex affect dynamics add limited information to the prediction of psychological well-being. *Nat. Hum. Behav.* 3 478–491. 10.1038/s41562-019-0555-0 30988484

[B11] Demiralp E. Thompson R. J. Mata J. Jaeggi S. M. Buschkuehl M. Barrett L. F. (2012). Feeling blue or turquoise? Emotional differentiation in major depressive disorder. *Psychol. Sci.* 23 1410–1416. 10.1177/0956797612444903 23070307 PMC4004625

[B12] Eid M. Schneider C. Schwenkmezger P. (1999). Do you feel better or worse? The validity of perceived deviations of mood states from mood traits. *Eur. J. Pers.* 13 283–306. 10.1002/(SICI)1099-0984(199907/08)13:4<283::AID-PER341<3.0.CO;2-0

[B13] Enders C. K. Tofighi D. (2007). Centering predictor variables in cross-sectional multilevel models: a new look at an old issue. *Psychol. Methods* 12 121–138. 10.1037/1082-989X.12.2.121 17563168

[B14] Erbas Y. Ceulemans E. Boonen J. Noens I. Kuppens P. (2013). Emotion differentiation in autism spectrum disorder. *Res. Autism Spectr. Disord.* 7 1221–1227. 10.1016/j.rasd.2013.07.007

[B15] Erbas Y. Ceulemans E. Kalokerinos E. K. Houben M. Koval P. Pe M. L. (2018). Why I don’t always know what I’m feeling: the role of stress in within-person fluctuations in emotion differentiation. *J. Pers. Soc. Psychol.* 115 179–191. 10.1037/pspa0000126 30024239

[B16] Erbas Y. Ceulemans E. Lee Pe M. Koval P. Kuppens P. (2014). Negative emotion differentiation: its personality and well-being correlates and a comparison of different assessment methods. *Cogn. Emot.* 28 1196–1213. 10.1080/02699931.2013.875890 24410047

[B17] Erbas Y. Kalokerinos E. K. Kuppens P. van Halem S. Ceulemans E. (2021). Momentary emotion differentiation: the derivation and validation of an index to study within-person fluctuations in emotion differentiation. *Assessment.* 10.1177/1073191121990089 [Epub ahead of print]. 33522259

[B18] Garde A. H. Albertsen K. Persson R. Hansen A. M. Rugulies R. (2011). Bi-directional associations between psychological arousal, cortisol, and sleep. *Behav. Sleep Med.* 10 28–40. 10.1080/15402002.2012.636272 22250777

[B19] Geldhof G. J. Preacher K. J. Zyphur M. J. (2014). Reliability estimation in a multilevel confirmatory factor analysis framework. *Psychol. Methods* 19 72–91. 10.1037/a0032138 23646988

[B20] Goldstein H. Browne W. Rasbash J. (2002). Partitioning variation in multilevel models. *Underst. Stat.* 1 223–231. 10.1207/S15328031US0104_02

[B21] Gräfe K. Zipfel S. Herzog W. Löwe B. (2004). Screening psychischer Störungen mit dem “Gesundheitsfragebogen für Patienten (PHQ-D)” [Screening for psychiatric disorders with the patient health questionnaire (PHQ)]. *Diagnostica* 50 171–181. 10.1026/0012-1924.50.4.171

[B22] Grommisch G. Koval P. Hinton J. D. X. Gleeson J. Hollenstein T. Kuppens P. (2020). Modeling individual differences in emotion regulation repertoire in daily life with multilevel latent profile analysis. *Emotion* 20 1462–1474. 10.1037/emo0000669 31478725

[B23] Hayes A. F. (2009). Beyond Baron and Kenny: statistical mediation analysis in the new millennium. *Commun. Monogr.* 76 408–420. 10.1080/03637750903310360

[B24] Hayes A. F. Rockwood N. J. (2020). Conditional process analysis: concepts, computation, and advances in the modeling of the contingencies of mechanisms. *Am. Behav. Sci.* 64 19–54. 10.1177/0002764219859633

[B25] Hox J. J. Moerbeek M. van de Schoot R. (2018). *Multilevel Analysis: Techniques and Applications. Quantitative Methodology Series*, 3rd Edn. New York, NY: Routledge.

[B26] Huffziger S. Ebner-Priemer U. Zamoscik V. Reinhard I. Kirsch P. Kuehner C. (2013). Effects of mood and rumination on cortisol levels in daily life: an ambulatory assessment study in remitted depressed patients and healthy controls. *Psychoneuroendocrinology* 38 2258–2267. 10.1016/j.psyneuen.2013.04.014 23684479

[B27] Kalokerinos E. K. Erbas Y. Ceulemans E. Kuppens P. (2019). Differentiate to regulate: low negative emotion differentiation is associated with ineffective use but not selection of emotion-regulation strategies. *Psychol. Sci.* 30 863–879. 10.1177/0956797619838763 30990768

[B28] Kashdan T. B. Barrett L. F. McKnight P. E. (2015). Unpacking emotion differentiation. *Curr. Dir. Psychol. Sci.* 24 10–16. 10.1177/0963721414550708

[B29] Kashdan T. B. Farmer A. S. (2014). Differentiating emotions across contexts: comparing adults with and without social anxiety disorder using random, social interaction, and daily experience sampling. *Emotion* 14 629–638. 10.1037/a0035796 24512246 PMC4191833

[B30] Kashdan T. B. Ferssizidis P. Collins R. L. Muraven M. (2010). Emotion differentiation as resilience against excessive alcohol use: an ecological momentary assessment in underage social drinkers. *Psychol. Sci.* 21 1341–1347. 10.1177/0956797610379863 20696854

[B31] Kimhy D. Vakhrusheva J. Khan S. Chang R. W. Hansen M. C. Ballon J. S. (2014). Emotional granularity and social functioning in individuals with schizophrenia: an experience sampling study. *J. Psychiatric Res.* 53 141–148. 10.1016/j.jpsychires.2014.01.020 24561000 PMC4000561

[B32] Klaperski S. von Dawans B. Heinrichs M. Fuchs R. (2013). Does the level of physical exercise affect physiological and psychological responses to psychosocial stress in women? *Psychol. Sport Exerc.* 14 266–274. 10.1016/j.psychsport.2012.11.003

[B33] Könen T. Dirk J. Schmiedek F. (2015). Cognitive benefits of last night’s sleep: daily variations in children’s sleep behavior are related to working memory fluctuations. *J. Child Psychol. Psychiatry* 56 171–182. 10.1111/jcpp.12296 25052368

[B34] Konjarski M. Murray G. Lee V. V. Jackson M. L. (2018). Reciprocal relationships between daily sleep and mood: a systematic review of naturalistic prospective studies. *Sleep Med. Rev.* 42 47–58. 10.1016/j.smrv.2018.05.005 30404728

[B35] Koval P. Brose A. Pe M. L. Houben M. Erbas Y. Champagne D. (2015). Emotional inertia and external events: the roles of exposure, reactivity, and recovery. *Emotion* 15 625–636. 10.1037/emo0000059 25844974

[B36] Leiner D. J. (2020). *SoSci Survey (Version 3.2.12) [Computer software].* München: SoSci Survey GmbH.

[B37] Lennarz H. K. Lichtwarck-Aschoff A. Timmerman M. E. Granic I. (2018). Emotion differentiation and its relation with emotional well-being in adolescents. *Cogn. Emot.* 32 651–657. 10.1080/02699931.2017.1338177 28602148

[B38] Lischetzke T. Pfeifer H. Crayen C. Eid M. (2012). Motivation to regulate mood as a mediator between state extraversion and pleasant–unpleasant mood. *J. Res. Pers.* 46 414–422. 10.1016/j.jrp.2012.04.002

[B39] Liu D. Y. Gilbert K. E. Thompson R. J. (2020). Emotion differentiation moderates the effects of rumination on depression: a longitudinal study. *Emotion* 20 1234–1243. 10.1037/emo0000627 31246044 PMC6933110

[B40] MacCann C. Erbas Y. Dejonckheere E. Minbashian A. Kuppens P. Fayn K. (2020). Emotional intelligence relates to emotions, emotion dynamics, and emotion complexity. *Eur. J. Psychol. Assess.* 36 460–470. 10.1027/1015-5759/a000588

[B41] MacKinnon D. P. Fairchild A. J. (2009). Current directions in mediation analysis. *Curr. Dir. Psychol. Sci.* 18 16–20. 10.1111/j.1467-8721.2009.01598.x 20157637 PMC2821103

[B42] MacKinnon D. P. Krull J. L. Lockwood C. M. (2000). Equivalence of the mediation, confounding and suppression effect. *Prev. Sci.* 1 173–181. 10.1023/A:1026595011371 11523746 PMC2819361

[B43] Mathieu J. E. Aguinis H. Culpepper S. A. Chen G. (2012). Understanding and estimating the power to detect cross-level interaction effects in multilevel modeling. *J. Appl. Psychol.* 97 951–966. 10.1037/a0028380 22582726

[B44] Meade A. W. Craig S. B. (2012). Identifying careless responses in survey data. *Psychol. Methods* 17 437–455. 10.1037/a0028085 22506584

[B45] Morin C. M. Rodrigue S. Ivers H. (2003). Role of stress, arousal, and coping skills in primary insomnia. *Psychosom. Med.* 65 259–267. 10.1097/01.psy.0000030391.09558.a3 12651993

[B46] Muthén B. (2010). *Bayesian Analysis in Mplus: A Brief Introduction.* Available online at: www.statmodel.com/download/IntroBayesVersion%203.pdf (accessed October 27, 2020).

[B47] Muthén L. K. Muthén B. (1998-2020). *Mplus User’s Guide*, 8th Edn. Los Angeles, CA: Muthén & Muthén.

[B48] Neubauer A. B. Scott S. B. Sliwinski M. J. Smyth J. M. (2020). How was your day? Convergence of aggregated momentary and retrospective end-of-day affect ratings across the adult life span. *J. Pers. Soc. Psychol.* 119 185–203. 10.1037/pspp0000248 31070397 PMC6842033

[B49] O’Toole M. S. Renna M. E. Elkjær E. Mikkelsen M. B. Mennin D. S. (2020). A systematic review and meta-analysis of the association between complexity of emotion experience and behavioral adaptation. *Emot. Rev.* 12 23–38. 10.1177/1754073919876019

[B50] Oh V. Y. S. Tong E. M. W. (2020). Negative emotion differentiation and long-term physical health-the moderating role of neuroticism. *Health Psychol.* 39 127–136. 10.1037/hea0000809 31556656

[B51] Ottenstein C. (2020). Emotion regulation effectiveness accounts for the associations of self-reported emotion differentiation with well-being and depression. *Cogn. Emot.* 34 994–1002. 10.1080/02699931.2019.1691506 31726942

[B52] Ottenstein C. Lischetzke T. (2020). Development of a novel method of emotion differentiation that uses open-ended descriptions of momentary affective states. *Assessment* 27 1928–1945. 10.1177/1073191119839138 30947508 PMC7545652

[B53] Pond R. S. Kashdan T. B. DeWall C. N. Savostyanova A. Lambert N. M. Fincham F. D. (2012). Emotion differentiation moderates aggressive tendencies in angry people: a daily diary analysis. *Emotion* 12 326–337. 10.1037/a0025762 22023359

[B54] Preacher K. J. Zhang Z. Zyphur M. J. (2016). Multilevel structural equation models for assessing moderation within and across levels of analysis. *Psychol. Methods* 21 189–205. 10.1037/met0000052 26651982

[B55] Preacher K. J. Zyphur M. J. Zhang Z. (2010). A general multilevel SEM framework for assessing multilevel mediation. *Psychol. Methods* 15 209–233. 10.1037/a0020141 20822249

[B56] Puterman E. DeLongis A. Pomaki G. (2010). Protecting us from ourselves: social support as a buffer of trait and state rumination. *J. Soc. Clin. Psychol.* 29 797–820. 10.1521/jscp.2010.29.7.797

[B57] Scherer K. R. (2005). What are emotions? And how can they be measured? *Soc. Sci. Inf.* 44 695–729. 10.1177/0539018405058216

[B58] Schreuder M. J. Wichers M. Hartman C. A. Menne-Lothmann C. Decoster J. van Winkel R. (2020). Lower emotional complexity as a prospective predictor of psychopathology in adolescents from the general population. *Emotion.* 10.1037/emo0000778 [Epub ahead of print]. 32658508

[B59] Seah T. H. S. Coifman K. G. (2021). Emotion differentiation and behavioral dysregulation in clinical and nonclinical samples: a meta-analysis. *Emotion* 10.1037/emo0000968 [Epub ahead of print]. 34264705

[B60] Spitzer R. L. Kroenke K. Williams J. B. (1999). Validation and utility of a self-report version of PRIME-MD: the PHQ primary care study. Primary care evaluation of mental disorders. Patient Health Questionnaire. *JAMA* 282 1737–1744. 10.1001/jama.282.18.1737 10568646

[B61] Starr L. R. Hershenberg R. Li Y. I. Shaw Z. A. (2017). When feelings lack precision: low positive and negative emotion differentiation and depressive symptoms in daily life. *Clin. Psychol. Sci.* 5 613–631. 10.1177/2167702617694657

[B62] Starr L. R. Hershenberg R. Shaw Z. A. Li Y. I. Santee A. C. (2020). The perils of murky emotions: emotion differentiation moderates the prospective relationship between naturalistic stress exposure and adolescent depression. *Emotion* 20 927–938. 10.1037/emo0000630 31246045 PMC6933107

[B63] Suvak M. K. Litz B. T. Sloan D. M. Zanarini M. C. Barrett L. F. Hofmann S. G. (2011). Emotional granularity and borderline personality disorder. *J. Abnorm. Psychol.* 120 414–426. 10.1037/a0021808 21171723 PMC3104325

[B64] Szymusiak R. McGinty D. (2008). Hypothalamic regulation of sleep and arousal. *Ann. N. Y. Acad. Sci.* 1129 275–286. 10.1196/annals.1417.027 18591488

[B65] Timm C. Ubl B. Zamoscik V. Ebner-Priemer U. Reinhard I. Huffziger S. (2017). Cognitive and affective trait and state factors influencing the long-term symptom course in remitted depressed patients. *PLoS One* 12:e0178759. 10.1371/journal.pone.0178759 28575049 PMC5456349

[B66] Tong E. M. W. Keng S.-L. (2017). The relationship between mindfulness and negative emotion differentiation: a test of multiple mediation pathways. *Mindfulness* 8 933–942. 10.1007/s12671-016-0669-7

[B67] Tousignant O. H. Taylor N. D. Suvak M. K. Fireman G. D. (2019). Effects of rumination and worry on sleep. *Behav. Ther.* 50 558–570. 10.1016/j.beth.2018.09.005 31030873

[B68] Trull T. J. Ebner-Priemer U. W. (2014). The role of ambulatory assessment in psychological science. *Curr. Dir. Psychol. Sci.* 23 466–470. 10.1177/0963721414550706 25530686 PMC4269226

[B69] Trull T. J. Ebner-Priemer U. W. (2020). Ambulatory assessment in psychopathology research: a review of recommended reporting guidelines and current practices. *J. Abnorm. Psychol.* 129 56–63. 10.1037/abn0000473 31868388

[B70] Tugade M. M. Fredrickson B. L. Barrett L. F. (2004). Psychological resilience and positive emotional granularity: examining the benefits of positive emotions on coping and health. *J. Pers.* 72 1161–1190. 10.1111/j.1467-6494.2004.00294.x 15509280 PMC1201429

[B71] Winzeler K. Voellmin A. Schäfer V. Meyer A. H. Cajochen C. Wilhelm F. H. (2014). Daily stress, presleep arousal, and sleep in healthy young women: a daily life computerized sleep diary and actigraphy study. *Sleep Med.* 15 359–366. 10.1016/j.sleep.2013.09.027 24503474

[B72] Zyphur M. J. Oswald F. L. (2015). Bayesian estimation and inference. *J. Manag.* 41 390–420. 10.1177/0149206313501200

